# A feasibility randomised controlled trial of extended brief intervention for alcohol misuse in adults with mild to moderate intellectual disabilities living in the community; The EBI-LD study

**DOI:** 10.1186/s13063-017-1953-0

**Published:** 2017-05-12

**Authors:** Christos Kouimtsidis, Alessandro Bosco, Katrina Scior, Gianluca Baio, Rachael Hunter, Vittoria Pezzoni, Eileen Mcnamara, Angela Hassiotis

**Affiliations:** 1 iHEAR, Surrey and Borders Partnership NHS Foundation Trust, 1 Prince Regent Road, London, TW3 1NE UK; 2 Division of Psychiatry, University College London, Maple House, 149 Tottenham Court Road, W1T 7NF London, UK; 3 Research Department of Clinical, Educational and Health Psychology, University College London, 1-19 Torrington Place, WC1E 6BT London, UK; 4 Department of Statistical Science, University College London, 1-19 Torrington Place, London, WC1E 6BT UK; 5 Department of Primary Care and Population Health Research, University College London, Royal Free Hospital Rowland Hill Street, NW3 2PF London, UK; 6 Hertfordshire Partnership NHS Foundation Trust, Woodside Road, Abbots Langley, Watford, WD5 0HT UK; 7 Barnet, Enfield and Haringey Mental Health NHS Trust, Barnet House 1255 High Road, London, N20 0EJ UK

**Keywords:** Alcohol misuse, Intellectual disabilities, Brief intervention

## Abstract

**Background:**

Extended brief interventions (EBIs) are effective in targeting alcohol misuse in the general population. However, little is known of the effects of EBI in adults with intellectual (also known as learning) disabilities. In this feasibility trial we compared EBI with usual care for alcohol misuse in adults with mild to moderate Intellectual Disability (ID).

**Methods:**

The study took place in three community ID networks of services in England. Participants aged 18–65 years with reported alcohol problems, a score ≥8 on the Alcohol Use Disorder Identification Test (AUDIT), and IQ <70 (+/5%CI) were recruited and were randomly allocated to either EBI (five weekly sessions and one follow-up at 8 weeks) and usual care or usual care alone. Research assistants were blind to arm allocation. Research assessments took place at baseline, 2 and 3 months. The primary outcome was reduction in alcohol consumption measured by the AUDIT. Preliminary health economic analysis was performed to investigate the costs of delivering EBI and the feasibility of a cost-effectiveness analysis in a full trial. The trial is closed.

**Results:**

Participants were recruited from January 2014 to August 2015. Thirty individuals were randomised (15 in each arm) and provided primary outcome data. In regard to harmful drinking, at baseline, all the participants exceeded the relevant threshold. At 8 weeks, the proportion of participants with harmful drinking had decreased to 60% for both groups, and at 12 weeks it had decreased by 66°7% and 46°7% for the intervention and the control groups, respectively. The unit cost for the delivery of EBI is £430.

**Conclusions:**

Recruitment to this trial has been proven challenging as prevalence of alcohol misuse in the targeted population was lower than anticipated. EBI may provide an effective low-intensity treatment for this population. Participants’ and carers’ feedback on their experience was overall positive. Further work needs to be undertaken to ascertain the group of participants that should be participating in a future definitive trial.

**Trial registration:**

Psychological Intervention Alcohol Misuse Learning Disability; isrctn.com, identifier:10.1186/ISRCTN58783633. Registered on 17 December 2013.

**Electronic supplementary material:**

The online version of this article (doi:10.1186/s13063-017-1953-0) contains supplementary material, which is available to authorized users.

## Background

Individuals with Intellectual Disability (ID) experience significant life-long deficits in intellectual functioning and adaptive behaviour [1].

Following the closure of long-stay hospitals, adults with ID have increasingly lived more independently in the community [2]. This has increased their exposure to environmental stressors, substance misuse [3] and alcohol misuse [4]. Prevalence of alcohol misuse is reported to be from as low as 0.5–2.5% [5] to as high as 22.5% [6] against a prevalence rate of 25% and 15% in men and women, respectively, in the general population [7].

Research suggests that alcohol misuse is experienced by nearly 50% of adults with ID who are drinkers [8] and that this negatively impacts on their functioning, relationships, physical and mental health, and safety [5, 8].

There is substantial literature on brief interventions (BIs) and extended brief interventions (EBIs) targeting alcohol misuse in the general population [9]. In contrast, the existing literature on adults with ID is limited in quantity and quality. In a systematic review of alcohol-related interventions, the authors found only one randomised controlled trial (RCT) evaluating educational materials about substance misuse in groups of adults with ID recruited from both community and hospital settings [10].

In terms of quality, the existing studies have used uncontrolled designs, pre and post evaluations or case studies/case series and or offenders in secure hospitals who are unlikely to use any skills learned in the community [10]. None of the studies has included an evaluation of cost-effectiveness.

Overall, the limited literature that is available suggests that motivational interviewing and education on the effects of excessive drinking using accessible materials are key elements in treatment programmes for adults with ID [10–12]. In general, interventions for mental disorders that are effective in other population groups may also be delivered to adults with ID, e.g. cognitive behaviour therapy (CBT), but adaptations in terms of session duration, treatment content, carer participation, and therapist delivery are necessary [13].

### Objectives

As a result of the paucity in evaluations of interventions for adults with ID living in the community, the present study aims to: (1) develop an adapted manualised extended BI for adults with ID and (2) test the feasibility of the intervention and assess, through qualitative interviews with participants and their carers, the perceived acceptability and usefulness of the intervention.

## Methods

### Trial design

The study design is reported in detail elsewhere [14]. This is a single-blind, parallel, two-arm, feasibility RCT comparing the clinical and cost-effectiveness of EBI and usual care against usual care only in reducing harmful drinking in adults with mild to moderate ID living in the community. The trial follows the guidelines of phase 2 of the Medical Research Council (MRC) for complex interventions [15] and includes: adaptation of the intervention, feasibility study including health economics, and process evaluation.

#### Assessments

Participant and family/paid carer assessments were conducted at baseline and at 2 (Alcohol Use Disorder Identification Test (AUDIT) only) [16] and 3 months by the research assistants (RAs) who were trained in assessing capacity and obtaining consent, promoting the study, administering the questionnaires, and interviewing participants with ID.

Fifty per cent of participants in the intervention group were interviewed about their experience of receiving EBI. A purposive sample of participants were recruited from all sites, including those with poor engagement or who had dropped out altogether. Interviews were conducted approximately 3 weeks after treatment. Interviews were semistructured and covered: recruitment process, therapy sessions, perceived effectiveness of therapy, research assessments, and participant’s decision to discontinue treatment (if relevant). The study service user reference group members assisted with the interviews to facilitate participant openness.

Carers of the participants allocated to EBI were asked to complete a survey exploring their views on the same topics.

### Participants

#### Inclusion criteria

- Adults with mild to moderate ID aged 18 years and over referred by professionals as having alcohol problems
- A score ≥8 on the Alcohol Use Disorder Identification Test (AUDIT) [16]
- Participants have lived in the area for the last 12 months
- IQ <70. Participants’ IQ was assessed with the Wechsler Abbreviated Scale for Intelligence (WASI) [17] unless a previous assessment was available

#### Exclusion criteria

- Severe to profound ID as per WASI score (standardised score <40)
- Participants with a cumulative AUDIT score higher than 9 in response to questions 4, 5, and 6 which specifically assess alcohol dependence. EBI is not recommended for alcohol dependence
- No knowledge of English
- Having received interventions targeting alcohol misuse in the last 12 months or currently receiving them
- Poly-substance misuse including alcohol (e.g. cocaine, heroin)

### Study settings

Participants were recruited from three areas in England covering urban and semirural sites (areas 1 and 2) and inner London (area 3), from a wide network of services for people with ID.

### Interventions

The adaptation of the intervention (e.g. number of sessions, materials to use during the sessions) was informed by the study service user reference group and the existing literature [13]. To that effect, more and longer sessions were offered compared to the typical EBI recommended for the general population with a longer review session offered 3 weeks later. The overall duration of the intervention was 8 weeks as per previous studies [14, 18]. The adapted EBI manual described the intervention and the content of the five weekly 30-min sessions and the 1-h follow-up session 3 weeks later. Motivational enhancement therapy (MET) as described in the UK Alcohol Treatment Trial (UKATT) MET manual was used in the first three sessions [18]. The content of sessions 4 and 5 was adapted from a CBT manual [19]. An information leaflet on the intervention manual was given to carers. Session 1 aimed to build a therapeutic relationship; while sessions 2 to 5 were ‘treatment’ sessions and session 6 was a review session. The treatment sessions were piloted with two service users and found to be suitable for delivery as planned.

Participants in the control group received usual care comprising various therapeutic interventions (e.g. talking therapy for generic coping skills, pharmacotherapy for comorbid mental disorders, nursing, psychology, and social care), with simple advice to modify their drinking.

### Outcomes

#### Proposed primary outcome measures for full trial

The AUDIT was administered to investigate changes in alcohol consumption. An easy-read AUDIT form was developed with illustrated response sheets to assist participants with recall (available on request by the authors). The language of the questionnaire was adapted and pictures were suggested by the RA and an independent from the study clinician working in ID (step 1). The easy-read version was then piloted with three service users with ID (two of whom were known to misuse alcohol) (step 2). Suggestions were incorporated and then the resulting version was reviewed by the two service users on the Steering Committee (step 3). Further suggestions regarding pictures were made and incorporated. The resulting version was finally piloted and feedback gathered from two members of the service user group ‘Can you hear us?’ (step 4). Suggestions were made on pictures and response scales.

The percentage of days of abstinence (PDAS) and percentage of days of heavy drinking (PDHD) were initially considered to be tested as potential primary outcomes for a future RCT, as suggested by the literature for the general population. Given though the complexity and the length of the interview required in order to assess those outcomes, and following discussion with the members of the study reference group it was decided to use the AUDIT-C score as an alternative primary outcome. AUDIT-C is a short version of the AUDIT and includes three questions assessing frequency of drinking, amount of drinking, and frequency of binge drinking. The definition of binge drinking was based on previous literature [20] and on sex-specific thresholds (men >8 units per day or >50 units per week; women >6 units per day or >35 units per week).

#### Secondary outcome measures

The following questionnaires were used and are described in more detail elsewhere [14]:

- Readiness to Change Questionnaire (RCQ), [21] measuring participants’ stage of change for excessive drinking. The questionnaire was made accessible through illustrations, simplified language, and a response sheet depicting ‘thumbs up’ and ‘thumbs down’ indicating ‘yes/no’ responses, respectively. Similar methodology using the same four steps to the one used for the development of the easy-read version of AUDIT was used for the RCQ. Language was simplified, pictures were suggested and the Likert scale changed to a Visual Analogue Scale
- Euro-QoL EQ-5D Youth (EQ-5D-Y) [22] and quality-adjusted life years (QALYs). QALYs have been calculated using the EQ-5D-3 L tariff as there are no value sets for the EQ-5D-Y. Adults with ID find the EQ-5D-3 L challenging to understand. Instead, we have used the EQ-5D-Y because of the simpler language it uses
- Clinical Outcomes in Routine Evaluation (CORE-LD) [23]
- Client Service Receipt Inventory (CSRI) to assess the feasibility of collecting patient reported service use [24]

#### Process evaluation – Treatment fidelity

- A self-rated checklist assessing the therapist’s own reflection of treatment delivery was scored after every session and it was used during supervision
- At least 10% of audio-taped therapy sessions (from all sessions and therapists) were rated by CK. A modified version of the Yale Adherence and Competence Scale, version II (YACS II) was used [25]. This is a widely used tool that assesses the frequency and intensity of sessions and how well techniques of MET and CBT are used, with a score from 1 to 6

### Sample size

No formal sample size calculation was carried out as this was a feasibility study. We set a sample size of 50 as sufficient to allow assessment of key objectives related to recruitment, retention, willingness to be randomised, data capture, completion rates of outcome measures, mean and standard deviation of primary outcome, and acceptability of the intervention [26].

### Randomisation and masking

Participants were randomly allocated to the intervention or control arm. Simple randomisation with an allocation ratio of 1:1 was used through a computer-generated code. All members of the research team (except CK) were blind to group allocation. Unmasking was monitored and researchers told participants not to divulge arm allocation at appointments. There was one incident of unmasking due to a delay in therapy initiation for medical reasons.

### Statistical and health economic analyses

The statistical methodology has been described elsewhere [14]. In brief, we compared dropout rates between the intervention and the control arms and we calculated descriptive statistics (means, medians, interquartile ranges, counts, and proportions, whenever relevant). All interval estimates are obtained by computing the 2.5% and the 97.5% quantiles of the sample data for the relevant variable. We did not perform statistical modelling (e.g. regression, confidence intervals, etc.) on the observed data and all our results presented below are meant to describe the output of the feasibility study, rather than be used to make inference about the general population.

With respect to the economic evaluation of EBI, we assessed the feasibility of gathering information for a cost-effectiveness analysis alongside a full RCT including testing the suitability of calculating quality-adjusted life years (QALYs) using the EQ-5D-Y.

The cost per patient of the intervention was calculated from data collected on therapist training, appointment duration and retention time for each appointment (see Additional file 1).

### Other analyses

Process evaluation of EBI included (1) assessment of implementation factors (training, supervision, fidelity, and sessions attended) as well as (2) mechanisms of impact, which was achieved through interviews with participants and a survey of their family and paid carers to explore the acceptability of the intervention [27]. The interviews were audio-recorded and analysed through directed content analysis, whereby the coding categories are created deductively [28]. A RA first read the transcripts and highlighted the sections that appeared to fit in the themes. For accuracy of coding, meetings with the research team then took place.

## Results

### Recruitment

**Fig. 1:**
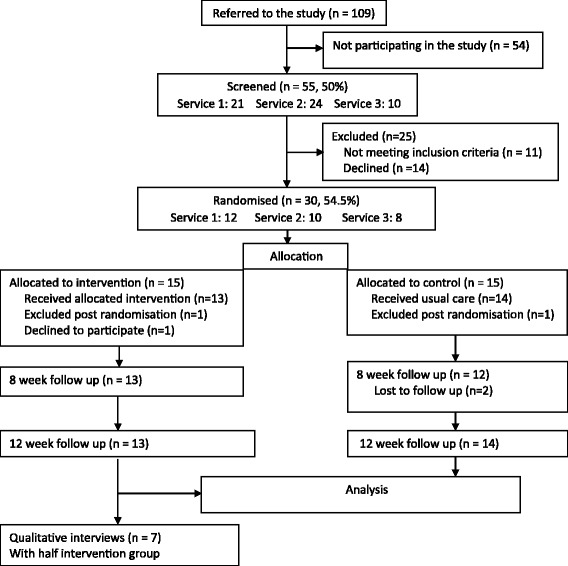
Consolidated Standards of Reporting Trials (CONSORT) diagram

Recruitment occurred from January 2014 to August 2015, with 3 months’ follow-up completed in November 2015 (see the Consolidated Standards of Reporting Trials (CONSORT) diagram in Fig. 1). Recruitment strategies used were based on those used in a previous study conducted by CK and VP suggesting a prevalence of alcohol misuse of 22.5%, hence the study was initially publicised only in the NHS participating services. Recruitment strategies were updated and modified according to feedback received by services and local staff to include direct recruitment from social clubs and supported accommodation, service databases for eligible participants with a history of alcohol misuse. Study promotion included phone calls, emails, letters, and presentations at meetings.

One hundred and nine adults with ID were referred to the study. Fifty-four participants were not screened due to a variety of reasons, such as not being interested in research (13), drinking was not a problem (11), being uncontactable (7), already receiving alcohol treatment (4), feeling unwell (2), poor English (2), having other drug problems (2), work commitment (1), and advised against participation by a carer (1). Eleven people did not give any reason.

Out of the 55 screened participants (50% of those referred), 25 were excluded from the study at randomisation due to being below (5) or above (3) AUDIT cut-off points (dependence questions only, as described in the ‘Exclusion criteria’ section), acute mental ill health (1), declined to participate (14) and being uncontactable (2). Thirty individuals (54.5% of those screened) were randomised and equally allocated to the intervention or control arm (see Table 1 for demographics and baseline scores). This gives an overall referral/randomisation ratio of 27.5%. Two participants were excluded after randomisation (one from each arm); one started alcohol treatment and the other was admitted to hospital for mental health treatment. A third participant withdrew consent. No harmful effects were reported.

**Table 1:** Participant demographic and baseline clinical details

Sociodemographic	Intervention arm (*N* = 15)	Control arm (*N* = 15)
	*N* (%)	*N* (%)
Age (years; median IQR)	45 (8–5)	44 (22.5)
Gender (M)	10 (66.6%)	10 (66.6%)
Ethnic origin		
White	12 (80%)	15 (100%)
Other	3 (20%)	-
ID level		
Moderate	6 (40%)	2 (13%)
Mild	9 (60%)	13 (86%)
Living circumstances		
Living alone	10 (66.6%)	8 (53.3%)
In family home with parents	2 (13.4%)	3 (20%)
With others	3 (20%)	4 (26.7%)
Clinical status		
Physical health problems		
Sensory problems	6 (40%)	3 (20%)
Mobility problems	1 (0.6%)	2 (1.2%)
Incontinence problems	2 (1.2%)	-
Study instruments	Mean (SD)	Mean (SD)
AUDIT score		
Baseline	22.13 (5.82)	20.46 (6.40)
8 weeks	15.00 (6.01)	15.08 (6.41)
12 weeks	14.50 (7.51)	16.57 (7.51)
RCQ score		
Precontemplation scale	−1.08 (1.44)	−0.57 (2.59)
Contemplation scale	1.50 (4.68)	3.00 (3.18)
Action scale	2.41 (4.71)	3.78 (3.28)
CORE-LD score		
Baseline	10.53 (7.37)	6.80 (4.55)
12 weeks	8.16 (5.70)	4.85 (4.20)
EQ-5D-Y		
Baseline	0.63 (0.39)	0.90 (0.12)
12 weeks	0.61 (0.36)	0.92 (0.12)
QALYs	0.14 (0.07)	0.21 (0.02)

Data listed are *N* (%), mean (SD), median interquartile range (IQR). The dash (-) is attributed when a score is zero. *AUDIT* Alcohol Use Disorders Identification Test, *CORE*-*LD* Clinical Outcomes in Routine Evaluation, *EQ-5D-Y* Euro-QoL EQ-5D Youth, *ID* Intellectual Disability, *QALYs* quality-adjusted life years, *RCQ* Readiness to Change Questionnaire,

Out of 30 participants, 25 (83%) completed the 8-week follow-up (2 excluded, 1 dropped out and 2 were unavailable) and 27 (90%) completed the 3-month follow-up (2 excluded, 1 dropped out).

### Primary outcome

**Fig. 2:**
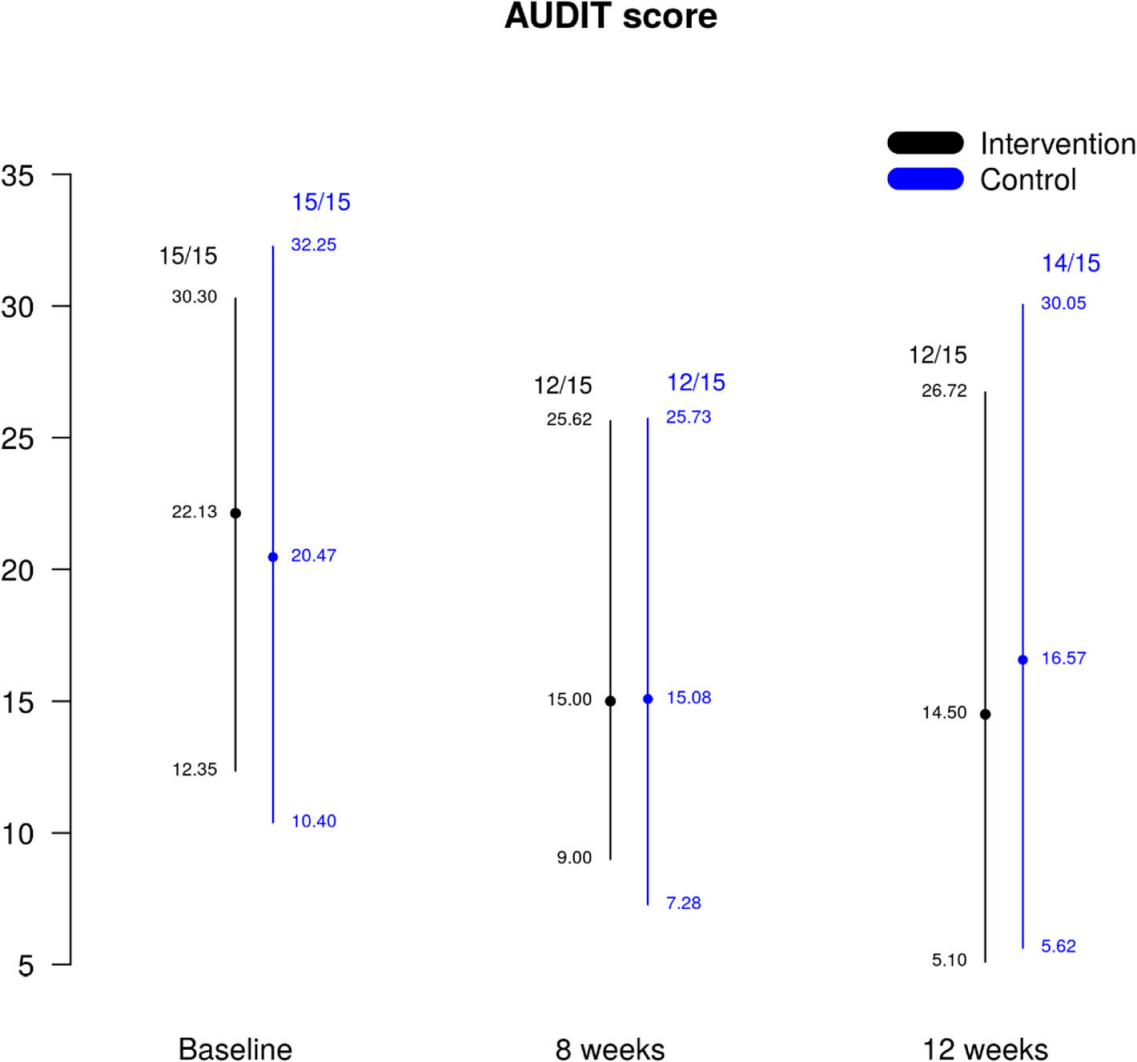
Alcohol Use Disorder Identification Test (AUDIT) score at each assessment time point

In this section we provide descriptive statistics for outcomes that are relevant for a full-scale trial. In regard to the primary outcome*,* both the intervention and the control groups show a potentially decreasing trend in the AUDIT score from baseline to the end of the 3-month follow-up (Fig. 2). Differences between groups and the first and second follow-up points appear less important. Given the small numbers, the estimates are associated with considerable uncertainty and the ranges presented in Fig. 2 do not show substantial differences across groups.

The lower threshold for ‘harmful drinking’ using the AUDIT is 8 for men and 5 for women. At baseline, all the participants exceeded the relevant threshold, but the proportion of participants exceeding the thresholds decreased over time: at 8 weeks, we noted a 60% decrease in both groups, while at 12 weeks the decrease was estimated at 66°7% for the intervention and at 46°7% for the treatment as usual (TAU) group. However, because the sample size is too small, variability in these estimates is too high to warrant any formal testing or comparison.

In considering the suitability of the primary outcome measure, we calculated the total score of the first three AUDIT questions (AUDIT-C). The intervention group has a rather sharp decrease in the value of the total AUDIT-C score (Q1 + Q2 + Q3) from 9.13 at baseline, to 7.58 at week 8, to 6.42 at week 12; this is in contrast with the control arm where the total score remains unchanged (8.87 to 8.00 to 8.93).

### Secondary outcomes

The analyses of secondary outcomes support moderate differences between the two arms. In regard to the RCQ scores, at follow-up a general decrease in the average score for the ‘Contemplation’ score was shown: in the intervention arm, the score reduced from 3.07 (95% confidence interval (CI) -6.60 to 8.00) at baseline to 1.50 (95% CI -5.72 to 6.72) at 12 weeks; in the usual care arm it reduced from 5.13 (95% CI -0.60 to 8.00) to 3.00 (95% CI -3.35 to 7.00). The usual care arm has slightly higher means and lower overall variability.

With respect to the CORE-LD, the intervention arm showed higher rates of psychological distress at baseline (score of 10.53, 95% CI 1.70 to 23.95) which reduced to 8.17 (95% CI 2.27 to 18.73) at 12 weeks. The corresponding values in the control group were 6.80 (95% CI 1.00 to 13.65) and of 4.86 (95% CI 1.00 to 13.02). Participants in both arms have shown improvement.

Descriptive statistics for the EQ-5D-Y tariff and QALYs are reported in Table 1. There was no significant relationship between AUDIT and EQ-5D-Y tariffs at baseline (Pearson’s correlation = -0°04, *p* = 0°84) or at 12 weeks (Pearson’s correlation = 0°07 *p* = 0°73).

The cost of the intervention is reported in Table 2. The costs are reported for two English National Health Service (NHS) agendas for change payment bands, reflecting different levels of experience, seniority, and responsibility. Therapists in the study were employed at bands 4 (therapy assistant) and 5 (qualified practitioner). The total cost per patient of the intervention is dependent on assumptions about the total number of patients treated per therapist trained. Assuming a caseload of 10 patients per therapist the cost per patient of EBI including training is £351 and £430 for band 4 and band 5 therapists, respectively. Completion of the CSRI and descriptive statistics is included in Additional file 1.

**Table 2:** Cost of the intervention

	Band 4	Band 5	Consultant	
Cost per hour	£29	£36	£107	
Training				
Face to face	2	2	2	(h)
Listening exercise	5	5	0	(h)
Total time	7	7	2	
Total cost	£203	£252	£214	
Assumed caseload	10	10	10	
Appointments				
Preparation before session	15	15		(min)
Travel	60	60		(min)
Session	33	33		(min)
Reflecting after session	15	15		(min)
Total time (min)	123	123		
Cost per appointment	£59.45	£73.80		
Average number of sessions	5.2	5.2		
Average cost of intervention				
Excluding training	£309.14	£383.76		
Including training	£350.84	£430.36	£21.40	

### Process evaluation

#### Resources and training

Four therapists delivered the intervention. They were assistant psychologists or equivalent, working in ID services in the NHS, having had basic training in delivering psychological interventions as part of their doctorate training. They were supervised by CK who also delivered a 1-day training on motivational interviewing and the use of the manual.

#### Fidelity

Forty-three out of 68 sessions were audio-recorded and 32 (47%) were scored by CK using the YACS II. The frequency/intensity of techniques used had an average score of 4.73 (3.0 to 6.2) with only five sessions scoring below 4.0, the acceptable cut-off. The average score for how well techniques were implemented was 5.0 (3.1 to 6.3) with only three scoring below 4.

#### Sessions attended

Thirteen out of 15 participants began therapy, (one excluded post randomisation and one declined treatment) and attended a mean of five sessions (range two to six). Nine participants attended all six sessions, one attended five, one three sessions and two attended only two sessions.

#### Participant experience of EBI-ID

Seven participants (six men and one woman), whose ages ranged from 39 to 70 years (mean = 49°7), were interviewed. Five have completed all sessions, one had declined treatment and another attended four sessions. The following themes were identified: being part of the research project, having therapy sessions, impact of therapy on drinking, reasons of dropping out from therapy.

##### Being part of the research project

With respect to ‘being part of the project’, six participants reported hearing about the study from an ID professional and one from a friend from church. When asked about the reason for their referral, five participants thought that this was related to their alcohol misuse. Other reasons included wanting to help others or feeling ‘down’. When asked whether they had received any help prior to the study, only one participant reported having attended Alcoholics Anonymous.

The participants thought that the research assessments and the questionnaires were acceptable and no questions were singled out as unhelpful or inappropriate. The use of pictures during the assessment enhanced the understanding of participants:

‘Hmm, some were hard, some were easy…especially when you say how much and how often…’ P6

‘Made me think, ‘cos err you don’t know until you ask these questions.’ P6

‘I think the pictures helped.’ P5

##### Experience of the therapy sessions

Regarding the recollection of the content of therapy, three participants reported on the practical tasks that they were assigned during therapy, one reported on discussing what had happened during the week and two participants reported that it was difficult to recall specific events from therapy:

‘Got me some information where I had to write down. Had to answer a few questions, when would I drink…’ P6

‘She asked what I done in the week.’ P5

Participants’ views on the home assignments were mixed. Three participants reported negative views about these. As described by one participant, it made him feel ‘back to school’ P5. One participant viewed the homework positively:

‘Yeah… made me think…what I did, what I do from day to day… everything from getting up in the morning…’ P6

Regarding the perceived benefits experienced from the therapy, two participants reported on being able to talk freely about drinking problems with someone who is outside of their social network and one who has being listened to:

‘Erm… someone outside the family, someone independent.’ P6

‘It was helpful, the talking about it. Yeah it helped me yeah.’ P1

‘She was helpful, she did listen.’ P4

However, one participant reported problems in adapting to the communication style of the therapist and another participant felt that she feared that her carers could watch her record her alcohol intake:

‘To start with, she… talk very fast. But each meeting sort of slowed down…’ P6

‘People can see me using them (materials).’ P2

With respect to barriers to attending sessions, one participant reported being busy with other engagements as the greatest difficulty. Most participants reported that the number and length of sessions was adequate. One participant, however, thought that fewer sessions would be better:

‘It came at the wrong time for me really ‘cos I was looking after X [a friend], trying to do everything at once.’ P4

##### Impact of therapy on drinking

In regard to the perceived impact of therapy on drinking, a positive impact of therapy was reported by three participants. Two participants felt that there was no change, while two were unsure. One participant reported benefitting from therapy at first, but then found it difficult to maintain the positive change over time:

‘If I am “down” I can talk to any staff.’ P5

‘No…but I think more, I think do I need a pint or should I just get a half?’ P7

‘It was good therapy. It made me think about things, I can make my pint last 2 hours.’ P7

‘To start with it did…but then it sort of hmm, it’s like novelties… and before long I went back to square one carrying on…’ P6

##### Reasons for dropping out/declining to see a therapist

With respect to ‘dropping out/declining to see a therapist’, the participant who had declined to start therapy reported feeling hesitant about meeting a new person, whereas the participant who had dropped out after four sessions reported that his job made it difficult for him to make time to attend sessions.

#### Carers

The survey was completed by six carers (four paid carers and two family carers) and one health professional. Alcohol misuse was reported to be common among service users by three carers, uncommon by one carer and three felt unsure about the quantity of alcohol consumed by the service user. Six carers felt that the research assessments were easy/very easy to understand. One carer, however, felt that the service user struggled with some of the questions. Three carers felt positive about the length of the sessions, and one believed that the frequency was not high enough. Two carers of the service users who withdrew from therapy explained that the reasons were a negative impact of therapy, i.e. made the participant crave alcohol, and that therapy sessions increased psychological distress in the participant.

## Discussion

The present study has potentially important research and clinical implications. The dearth of evidence-based interventions for alcohol misuse in adults with ID may contribute to late access to treatment and may negatively impact the quality of care provided. This study assessed the feasibility of conducting an RCT to evaluate the clinical and cost benefits of EBI and usual care for adults with mild to moderate ID. To our knowledge, this study is the first to employ a design for complex interventions to adapt and test EBI in this population in the community or other settings (Additional file 2). The intervention is theory-based and uses both motivational and cognitive behavioural techniques to affect change in behaviour. In addition, it is a relatively low-intensity intervention that can be delivered by generic workers requiring minimal training but skilled supervision [29].

This being a feasibility study, the main aim was to assess parameters that will inform a future RCT study. The referral/screening ratio of this study (50%) was far lower that other studies of alcohol intervention in the general population (UKATT study 84.4%) [18]. To the contrary the screening/randomisation ratio (54.5%) was far higher than that of the UKATT study (27%) [18], giving an overall referral/randomisation ratio of 27.5% which is higher than that of the UKATT study (22.9%) [18]. Nine out of 15 participants (60%) allocated to the intervention have completed all six sessions, with a median attendance of five sessions, showing that the adherence to the intervention was very good. Furthermore, qualitative data suggested a positive experience of the research by both participants and carers.

With respect to the acceptability of the intervention, the participants reported gaining a good understanding of the consequences of alcohol misuse and of the strategies to avoid harmful drinking. Our participants reported that recording the quantity of alcohol consumed proved effective in reducing their intake. The lack of support from staff was reported as a barrier to homework completion which is a recognised difficulty in psychosocial interventions research [13].

One of the challenges encountered in this trial was the low number of cases identified and referred onto the study by local professionals. A previous study within the same treatment system identified a prevalence of 22.5% of harmful and dependent drinking [6]. To that effect, the initial recruitment strategy has targeted NHS ID specialist services. Despite the modification of the initial recruitment strategy to include recruitment from a wider variety of services and promotion methods, gains in number of participants were limited. An important barrier appeared to be the resistance of care staff. They felt that the study would add to their workload (a problem generic to clinical research) or that they had no contact with eligible participants (mostly in the third sector), possibly due to perceiving misuse of alcohol by service users as a professional failure to prevent it.

The recruitment challenges raise the question whether the prevalence of alcohol misuse in people with ID is indeed low or whether those who misuse alcohol are not diagnosed as having alcohol misuse. The recently published Adult Psychiatric Morbidity Survey 2014, reported a prevalence of harmful drinkers (AUDIT score 8–16) of 17.8% in men and 15.2% in women with low IQ (<84), thus giving weight to our starting hypothesis that alcohol misuse is a risk for people with ID living in the community [30]. It could be argued though that service users with ID living in staffed supported accommodation have less exposure to alcohol and are better supervised, hence protected. However, this realistic speculation could not be backed up by hard evidence from our data because of the limited sample size and the impact of missing data. Therefore, the question remains: ‘Where do the vulnerable people with ID live, what support do they have and what service would be in the best place to offer an intervention to help them change their drinking behaviour?’ To that effect, in view of considering a future definitive RCT of EBI, recruitment should encompass primary care transition services to include young people up to age 25 years with borderline intellectual functioning or mild ID who are more likely to be exposed to substance misuse and incorporate approaches to improve buy-in by support staff.

This being a feasibility study based on a small sample, we urge caution when interpreting our findings. The main aim was to test alternative potential primary outcomes, to be used in a future RCT. AUDIT and AUDIT-C were tested to that effect. For both the intervention and the control arms, we found a potential reduction in alcohol consumption in participants from baseline to 8 and 12 weeks. The identification of the best primary outcome measure to assess change in alcohol consumption and related behaviour in ID population remains a challenge. As stated by NICE guidelines (2011), this outcome instrument should be able to measure changes in drinking behaviour, while it is ‘feasible and implementable in routine clinical care’. They recommend AUDIT for case identification and initial assessment of problem severity [7]. Furthermore, according to NICE guidelines (2010), AUDIT is regarded as the ‘gold standard’ screening questionnaire for detecting hazardous and harmful drinking, which was the target population of this study [31].

The NICE guidelines (2011) [7] state that ‘the accuracy of the assessment of alcohol consumption from self-reported alcohol consumption can be enhanced by interviewing individuals who are not intoxicated, giving written assurances of confidentiality, encouraging openness and honesty, asking clearly worded questions and providing memory aids to recall drinking (such as drinking diaries)’ as recommended by Sobell and Sobell (2003) [32]. Although such an approach has been considered the gold standard in clinical research for the general population it has not been tested in populations with ID. The experience from the current study suggests that participants found the completion of drinking diaries (as a component of the intervention) challenging and support by carers was required. The literature on populations with ID reveals a paucity of measures in this area (as well as other areas of mental health in ID) and clearly there is a need for further development of appropriate assessment measures or adaptation of existing ones designed for the general population such as AUDIT [33].

Our findings from the economic evaluation assessing the suitability of the EQ-5D in EBI replicated findings from previous studies in adults without ID in that the EQ-5D is not correlated with the AUDIT and, hence, does not relate to levels of hazardous drinking [34].

### Lessons learned

This study of adapting and testing an alcohol intervention recommended for the general population in the ID population has taught us several lessons on conducting a future trial on this topic. Although we believe that we were able to respond to difficulties in recruitment as they arose, some more general points need to be considered: first, greater involvement of service users, family and paid carers in the design and execution of the research may increase understanding of the problem to be addressed in the study. Second, appropriate induction of researchers to aid recall in the participants and potentially including a carer-rated primary/secondary measure of the outcome of interest could provide an additional perspective. Health and social care professionals’ buy-in of the study is very important in order to enhance their understanding of the problem under investigation and to augment their support in identifying potential participants.

## Conclusions

It is a striking observation from a number of sources, including the recently published NICE guidelines on mental disorders in people with ID (NG54, 2016), that while there is extremely high prevalence, severity and complexity of need in this population, there is a disproportionate and woefully inadequate evidence base to recommend how to address the need.

Recruitment to this trial has proved challenging mostly due to the smaller than expected [6] number of cases identified by professionals and referred to the study. Primary care may need to be included as an option for participant identification, given that the population with mild to moderate ID most likely to be exposed to substances may not be under the care of specialist services and in their majority only receive minimal support. EBI for harmful alcohol use can be provided to this population and was well received by both participants and carers. However, its clinical and cost-effectiveness in this population group remains to be fully investigated in a future multicentre trial.

## Additional files

Additional file 1: Results of economic evaluation. (DOCX 27 kb)

Additional file 2: CONSORT Checklist. (DOCX 18 kb)

## Abbreviations

AUDIT: Alcohol Use Disorder Identification Test
BI: Brief interventions
CBT: Cognitive behaviour therapy
CORE-LD: Clinical Outcomes in Routine Evaluation in Learning Disabilities
CSRI: Client Service Receipt Inventory
EBI: Extended brief interventions
EQ-5D-Y: Euro-QoL EQ-5D Youth
ID: Intellectual Disability
MET: Motivational enhancement therapy
MRC: Medical Research Council
NHS: National Health Service
PDAS: Percentage of days of abstinence
PDHD: Percentage of days of heavy drinking
QALYs: Quality-adjusted life years
RCQ: Readiness to Change Questionnaire
RCT: Randomised controlled trial
WASI: Wechsler Abbreviated Scale for Intelligence
YACS II: Yale Adherence and Competence Scale, version II
