# Supplementary material for: A feasibility randomised controlled trial of extended brief intervention for alcohol misuse in adults with mild to moderate intellectual disabilities living in the community; The EBI-LD study
Source: Trials. 2017 May 12;18:216. doi: 10.1186/s13063-017-1953-0 (PMC5427589; doi:10.1186/s13063-017-1953-0)
Supplement: Supplementary file 1 — Results of economic evaluation. (DOCX 27 kb) [file 13063_2017_1953_MOESM1_ESM.docx]

Additional File 1

**Health care, social care and criminal justice resource use and costs**

**Additional File Table 1:** Mean resource use per patient (standard deviation (SD)) over 12 weeks at baseline and follow-up based on completion of the client service receipt inventory (CSRI) with the assistance of a researcher.

| **Follow-Up point** | **Baseline** | | **12 weeks** | |
| --- | --- | --- | --- | --- |
| **Trial Arm** | **Intervention arm** | **Control -Arm** | **Intervention arm** | **Control -Arm** |
| **Number of patients (N)** | 15 | 15 | 12 | 14 |
| **Community health and social care mean (SD)** |  |  |  |  |
| GP (surgery) | 2.7 (2.2) | 3.2 (2.5) | 0.7 (1.5) | 1.4 (0.93) |
| District Nurse | 1.1 (3.8) | 3.4 (8.1) | 0.6 (1.7) | 0.1 (0.5) |
| Mental health Nurse | 1.8 (5.3) | 0 | 0.3 (0.9) | 0.1 (0.4) |
| Learning disabilities nurse | 0.4 (1.5) | 0.5 (1.5) | 0.4 (1.0) | 0.2 (0.6) |
| Psychiatrist | 1.9 (3.7) | 0.7 (0.8) | 0.3 (0.6) | 0.1 (0.4) |
| Psychologist | 0.9 (3.1) | 1.7 (3.3) | 0 | 0.8 (2.2) |
| Social Worker | 3.5 (6.1) | 0.6 (1.1) | 0.7 (2.0) | 0.6 (0.8) |
| Occupational therapist | 0.1 (0.5) | 0.4 (1.6) | 0.3 (0.9) | 0 |
| Art/Drama/Music therapy | 0.1 (0.3) | 1.2 (6.5) | 0 | 0 |
| Counsellor | 0 | 1 (3.4) | 0 | 0.2 (0.8) |
| Physiotherapist | 0.2 (0.8) | 1.1 (0.8) | 0 | 0 |
| Dentist | 0.7 (1.1) | 0.7 (0.8) | 0.4 (0.9) | 0.6 (0.6) |
| Community support worker | 40.7 (62.3) | 21.5 (55.0) | 21.1 (32.6) | 25.5 (52.2) |
|  |  |  |  |  |
| **Inpatient (average nights)** |  |  |  |  |
| Mental Health | 6.1 (24) | 0 | 0 | 0 |
| Unplanned acute care | 0.9 (2.4) | 0.3 (0.8) | 0 | 0.6 (2.1) |
| Planned (elective) acute care | 0 | 0 | 1.3 (4.6) | 0 |
| Outpatient |  |  |  |  |
| Mental Health | 0.2 (0.4) | 0.3 (0.6) | 0.1 (0.3) | 0.1 (0.3) |
| Acute Care | 0.1 (0.4) | 0.4 (0.7) | 0 | 0.4 (1.3) |
| Day Case | 0.9 (1.8) | 0.3 (0.8) | 0 | 0.1 (0.4) |
|  |  |  |  |  |
| **A&E attendances** |  |  |  |  |
| Alcohol related | 0.3 (0.8) | 0.3 (0.5) | 0.2 (0.4) | 0.1 (0.4) |
| Not alcohol related | 0.1 (0.3) | 0.1 (0.4) | 0.1 (0.3) | 0 |
|  |  |  |  |  |
| **Criminal Justice** |  |  |  |  |
| Police contacts | 0.5 (0.8) | 0 | 0.1 (0.3) | 0 |
| Learning disabilities assessment | 0.1 (0.3) | 0 | 0 | 0 |
| Probation | 0.1 | 0 | 0 | 0.1 |

GP = General Practitioner; A&E = accident and emergency department.

**Additional File Table 2:** Unit costs for costing of health care, social care and criminal justice resource use. All costs are in 2014/2015 British Pounds.

| **Resource Use** | **Unit Cost (Per contact)** | **Source** |
| --- | --- | --- |
| **Community health and social care*** |  |  |
| GP (surgery) | 54 | PSSRU |
| District Nurse | 39 | PSSRU, Ball et al 2014 |
| Mental health Nurse | 17.5 | PSSRU, Ball et al 2014 |
| Learning disabilities nurse | 35 | PSSRU |
| Psychiatrist | 39 | PSSRU, Cruz et al 2013 |
| Psychologist | 44 | PSSRU, Pomerantz et al 2009 |
| Social Worker | 55 | PSSRU |
| Occupational therapist | 33 | PSSRU, Renforth et al 2014 |
| Art/Drama/Music therapy | 44 | PSSRU |
| Counsellor | 44 | PSSRU |
| Physiotherapist | 34 | PSSRU |
| Dentist | 71 | PSSRU |
| Community support worker | 51 | PSSRU |
| **Acute and specialist care** |  |  |
| Mental Health (per bed day) | 223 | PSSRU |
| Short non-elective stay | 608 | PSSRU |
| Long non-elective stay | 2863 | PSSRU |
| Planned (elective) acute care | 3405 | PSSRU |
| Mental Health (outpatient) | 107 | PSSRU |
| General medical outpatient | 112 | PSSRU |
| Day Case | 704 | PSSRU |
| A&E attendance (admitted) | 167 | Reference costs |
| A&E attendance (not admitted) | 108 | Reference costs |
| **Criminal Justice** |  |  |
| Police contact (arrested) | 267 | Heslin et al 2017 |
| Police contact (not-arrested) | 841 | Heslin et al 2017 |
| Learning disabilities assessment | 284 | PSSRU |
| Probation | 2690 | Hayhurst et al 2015 |

PSSRU = Personal and Social Services Research Unit. Curtis et al 2016.

GP = General Practitioner; A&E = accident and emergency department.

*Community health and social care unit costs are calculated based on hourly face to face costs from PSSRU multiplied by duration of appointment as specified in the references provided.

**Additional File Table 3:** Mean cost per patient (standard deviation (SD)) over 12 weeks at baseline and follow-up based on completion of the client service receipt inventory (CSRI) with the assistance of a researcher and unit costs reported in Additional File Table 2. All costs are in 2014/2015 British Pounds.

| **Follow-Up point** | **Baseline** | | **12 weeks** | |
| --- | --- | --- | --- | --- |
| **Trial Arm** | **Intervention arm** | **Control -Arm** | **Intervention arm** | **Control -Arm** |
| **Number of patients (N)** | 15 | 15 | 12 | 14 |
| **Community health and social care mean (SD)** |  |  |  |  |
| GP (surgery) | 147.60 (120) | 172.08 (137) | 36.00 (81) | 73.29 (50) |
| District Nurse | 41.60 (151) | 135.20 (314) | 22.75 (67) | 5.57 (20.85) |
| Mental health Nurse | 31.50 (92) | 0 | 4.38 (15) | 2.50 (6.35) |
| Learning disabilities nurse | 14 (54) | 18.67 (54) | 14.58 (35) | 7.50 (20.26) |
| Psychiatrist | 75.40 (143) | 26 (32) | 9.75 (24) | 5.57 (14) |
| Psychologist | 38.13 (136) | 76.27 (147) | 0 | 34.57 (98) |
| Social Worker | 190.67 (333.82) | 33 (58) | 36.67 (111) | 31.43 (41.58) |
| Occupational therapist | 4.40 (17) | 13.20 (51) | 8.25 (29) | 0 |
| Art/Drama/Music therapy | 2.93 (11) | 73.33 (284) | 0 | 0 |
| Counsellor | 0 | 44 (148) | 0 | 9.43 (35) |
| Physiotherapist | 6.80 (26) | 0 | 0 | 0 |
| Dentist | 52.07 (50) | 73.73 (56.72) | 29.58 (64) | 45.64 (45) |
| Community support worker | 2074 (3176) | 1078.80 (2810) | 1075 (1664) | 1301 (2664) |
| **Total Cost - community care** | **2679 (3203)** | **1763 (2796)** | **1237 (1653)** | **1516 (2631)** |
|  |  |  |  |  |
|  |  |  |  |  |
| **Inpatient mean cost (SD)** |  |  |  |  |
| Mental Health | 1353 (5240) | 0 | 0 | 0 |
| Unplanned acute care | 381 (1007) | 81 (214) | 0 | 205 (765) |
| Planned (elective) acute care | 0 | 0 | 284 (983) | 0 |
| Outpatient |  |  |  |  |
| Mental Health | 21 (44) | 28 (64) | 9 (30.9) | 8 (29) |
| Acute Care | 15 (39) | 45 (83) | 0 | 40 (150) |
| Day Case | 657 (1261) | 235 (575) | 0 | 101 (256) |
|  |  |  |  |  |
| A&E attendances mean cost (SD) |  |  |  |  |
| Alcohol related | 40 (102) | 41 (71) | 18 (42) | 24 (61) |
| Not alcohol related | 7.2 (28) | 18 (50) | 9 (31) | 0 |
|  |  |  |  |  |
| **Total acute care cost** | **2475 (5211)** | **448 (740)** | **320 (973)** | **377 (911)** |
|  |  |  |  |  |
| **Total Health Care Cost** | **5154 (6136)** | **2211 (2771)** | **1557 (1785)** | **1893 (2800)** |

|  |  |  |  |  |
| --- | --- | --- | --- | --- |
| **Criminal Justice** |  |  |  |  |
| Cost of police | 201 (397) | 0 | 22 (77) | 0 |
| Learning disabilities assessment | 19 (73) | 0 | 0 | 0 |
| Probation | 179 (695) | 0 | 0 | 192 (719) |
|  |  |  |  |  |
| **Total Criminal Justice Cost** | **399.4 (766)** | **0** | **22 (77)** | **192 (719)** |
|  |  |  |  |  |
| **Total all costs** | **5554 (6215)** | **2211 (2771)** | **1579 (1768)** | **2085 (2805)** |

GP = General Practitioner; A&E = accident and emergency department.

**References**

Ball, J., et al. (2014). "Survey of district and community nurses in 2013." Report to the Royal College of Nursing National Nursing Research Unit, King’s College London: London.

Cruz, M., et al. (2013). "Appointment length, psychiatrists’ communication behaviors, and medication management appointment adherence." Psychiatric Services.

Cutis, L. and A. Burns (2015). "Unit costs of health and social care." PSSRU: Personal Social Services Research Unit.

Hayhurst et al (2015). “The effectiveness and cost-effectiveness of diversion and aftercare programmes for offenders using class A drugs: a systematic review and economic evaluation.” Health Technology Assessment **19**(6): 1-198.

Heslin et al (2017) “Costs of the police service and mental healthcare pathways experienced by individuals with enduring mental health needs.” British Journal of Psychiatry **210**(2): 157-164.

Pomerantz, A. S., et al. (2009). "The challenge of integrated care for mental health: Leaving the 50 minute hour and other sacred things." Journal of Clinical Psychology in Medical Settings **16**(1): 40-46.

Renforth, P., et al. (2004). "Occupational therapy predischarge home visits: A study from a community hospital." The British Journal of Occupational Therapy **67**(11): 488-494.
