# Supplementary material for: A feasibility randomised controlled trial of extended brief intervention for alcohol misuse in adults with mild to moderate intellectual disabilities living in the community; The EBI-LD study
Source: Trials. 2017 May 12;18:216. doi: 10.1186/s13063-017-1953-0 (PMC5427589; doi:10.1186/s13063-017-1953-0)
Supplement: Supplementary file 2 — CONSORT Checklist. (DOCX 18 kb) [file 13063_2017_1953_MOESM2_ESM.docx]

| **Section/topic** | **Item number** | **Checklist item** | **Reported on page number** |
| --- | --- | --- | --- |
| **Title and abstract** | | |  |
| 1a | | Identification as a randomised trial in the title | 1 |
| 1b | | Structured summary of trial design, methods, results, and conclusions (for specific guidance see CONSORT for abstracts21,31) | 2 |
| **Introduction** | | |  |
| Background and objectives | 2a | Scientific background and explanation of rationale | 3 |
| 2b | | Specific objectives or hypotheses | 4 |
| **Methods** | | |  |
| Trial design | 3a | Description of trial design (such as parallel, factorial) including allocation ratio | 4 |
| 3b | | Important changes to methods after trial commencement (such as eligibility criteria), with reasons | NA |
| Participants | 4a | Eligibility criteria for participants | 5 |
| 4b | | Settings and locations where the data were collected | 6 |
| Interventions | 5 | The interventions for each group with sufficient details to allow replication, including how and when they were actually administered | 6 |
| Outcomes | 6a | Completely defined prespecified primary and secondary outcome measures, including how and when they were assessed | 7,8 |
| 6b | | Any changes to trial outcomes after the trial commenced, with reasons | 7 |
| Sample size | 7a | How sample size was determined | 8 |
| 7b | | When applicable, explanation of any interim analyses and stopping guidelines | NA |
| **Randomisation** | | |  |
| Sequence generation | 8a | Method used to generate the random allocation sequence | 8 |
| 8b | | Type of randomisation; details of any restriction (such as blocking and block size) | 8 |
| Allocation concealment mechanism | 9 | Mechanism used to implement the random allocation sequence (such as sequentially numbered containers), describing any steps taken to conceal the sequence until interventions were assigned | 9 |
| Implementation | 10 | Who generated the random allocation sequence, who enrolled participants, and who assigned participants to interventions | 9 |
| Blinding | 11a | If done, who was blinded after assignment to interventions (for example, participants, care providers, those assessing outcomes) and how | 9 |
| 11b | | If relevant, description of the similarity of interventions | NA |
| Statistical methods | 12a | Statistical methods used to compare groups for primary and secondary outcomes | 9 |
| 12b | | Methods for additional analyses, such as subgroup analyses and adjusted analyses | NA |
| **Results** | | |  |
| Participant flow (a diagram is strongly recommended) | 13a | For each group, the numbers of participants who were randomly assigned, received intended treatment, and were analysed for the primary outcome | 10 |
| 13b | | For each group, losses and exclusions after randomisation, together with reasons | 10 |
| Recruitment | 14a | Dates defining the periods of recruitment and follow-up | 10 |
| 14b | | Why the trial ended or was stopped | NA |
| Baseline data | 15 | A table showing baseline demographic and clinical characteristics for each group | 12 |
| Numbers analysed | 16 | For each group, number of participants (denominator) included in each analysis and whether the analysis was by original assigned groups | 12 |
| Outcomes and estimation | 17a | For each primary and secondary outcome, results for each group, and the estimated effect size and its precision (such as 95% CI) | 12, 13, 14 |
| 17b | | For binary outcomes, presentation of both absolute and relative effect sizes is recommended | NA |
| Ancillary analyses | 18 | Results of any other analyses performed, including subgroup analyses and adjusted analyses, distinguishing prespecified from exploratory | 14-19 |
| Harms | 19 | All important harms or unintended effects in each group (for specific guidance see CONSORT for harms28) | 11 |
| **Discussion** | | |  |
| Limitations | 20 | Trial limitations, addressing sources of potential bias, imprecision, and, if relevant, multiplicity of analyses | 19 |
| Generalisability | 21 | Generalisability (external validity, applicability) of the trial findings | 19 |
| Interpretation | 22 | Interpretation consistent with results, balancing benefits and harms, and considering other relevant evidence | 20 |
| **Other information** | | |  |
| Registration | 23 | Registration number and name of trial registry | 21 |
| Protocol | 24 | Where the full trial protocol can be accessed, if available | 21 |
| Funding | 25 | Sources of funding and other support (such as supply of drugs), role of funders | 21 |
